# Influence of embedding media on the accuracy of working length determination by means of apex locator: an ex vivo study

**DOI:** 10.1038/s41598-021-82942-6

**Published:** 2021-02-08

**Authors:** Thomas Gerhard Wolf, Anna Krauß-Mironjuk, Richard Johannes Wierichs, Benjamín Briseño-Marroquín

**Affiliations:** 1 Department of Restorative, Preventive and Pediatric Dentistry, School of Dental Medicine, University of Bern, Freiburgstrasse 7, 3010 Bern, Switzerland; 2 Department of Periodontology and Operative Dentistry, University Medical Center of the Johannes-Gutenberg-University Mainz, Mainz, Germany

**Keywords:** Electrophysiology, Experimental models of disease, Outcomes research, Medical research, Translational research, Biomaterials, Biomedical materials, Tissues, Oral anatomy, Dentistry

## Abstract

The aim of this research was to determine ex vivo the influence on accuracy of five different embedding media, for investigative and educational purposes, and one electronic apex locator. 110 human extracted mature roots of permanent single-rooted human teeth were used. The roots were embedded in alginate, stick sponge, 2% agar–agar and 6% and 12% gelatin. The actual working length to the physiological foramen was determined under a stereo-microscope (16 ×) and the electronic working lengths with the Elements Diagnostic Unit and a K-file ISO 10. The accuracy ranges of the accumulated measurements, when allowing a ± 0.5 mm tolerance, went from 98.2% (6% and 12% gelatin), 93.7% (alginate), 92.8% (2% agar–agar) to 91.7% (sponge). The exact measurements at the physiological foramen ranged from 80.0% (6% gelatin), 76.5% (2% agar–agar), 71.8% (12% gelatin), 68.2% (alginate) to 64.5% (sponge). Although relatively seldom (n = 24), measurements with deviations of more than ± 0.5 mm were also observed; thus, the accuracy of the working length determination results per se can be considered as clinically acceptable. The results of this research allow a recommendation of the investigated embedding media for electronic working length determination models for educational and research purposes in endodontics.

## Introduction

Assessment of the working length can be considered to be an imperative procedure during root canal preparation procedure and its accurate determination to be of the outmost importance for successful endodontic treatment^1,2^. The working length can be defined as the distance between the most coronal or incisal edge or cusp tip and an apical reference point given by the physiological foramen^3^. If the working length is underestimated, tissue residues and/or bacteria will remain in the non-instrumented areas of the root canal system. On the other hand, if the working length is determined beyond the apical boundaries, vital and/or infected material will be transported into the periapical tissues. An erroneously determined working length will most probably compromise the outcome of an endodontic treatment, as it will lead to shaping and filling procedures that are inaccurate. This can lead to periapical tissue inflammation and/or infection^1,2^. All possible measures should be undertaken to constrain mechanical procedures as well as chemicals and possible toxins from irritating materials within the root canal system, but not beyond the physiological foramen limits in order to minimize the risk of bacterial contamination and/or mechanical or chemical irritation of the peri-radicular tissues due to irrigating solutions, filling materials and over-instrumentation^4^. These precautions will enhance the success rate of an endodontic treatment^4^.

It has been reported^5,6^, in contrast with a report from Switzerland 25 years ago^7^, that in the last decade, a majority of operators surveyed determine the working length by means of an apex locator. However, although it has been reported in several in vitro studies^8–13^ that a solely electronic working length determination under different clinical conditions leads to clinically acceptable results, the actual guidelines of professional endodontic societies^14,15^ suggest that the working length should be determined electronically and subsequently substantiated by means of an X-ray image. Despite this fact, different research groups report^5,6^ that only approximately 50% of the surveyed operators routinely combine the electronic and radiological working length determination methods. This electronic and radiological combination method rationale is based in the remaining possible limitations that electronic apex locators still have^8^. In an ex vivo investigation, no statistical differences were reported between the radiologic working length determination (included as gold standard) and an electronic device^16^; however, it would be clinically advisable to keep in mind that the working length determination by means of a radiograph alone could lead to overestimation and unintentional over-enlargement of the physiological foramen^17^. If the ALARA principle (“as low [radiation] as reasonably achievable”) is routinely taken into consideration, a combined clinical strategic combination employment of an electronic device and radiograph during the working length determination will enhance the working length determination accuracy and, concomitantly, patient radiation exposure reduction^16,18^.

Regardless of the working length determination method employed, an accurate preparation boundary is of great significance to ensure endodontic success^4^. The accuracy of electronic apex locators has been evaluated with in vivo or in vitro research methods, whereas the precision of the working length determination depends on the device and/or type of irrigation employed rather than the pulp tissue status^19^. Moreover, most ex vivo or in vitro studies^8–10,12,13,16,20–25^ usually compare the accuracy of specific devices under different clinical conditions with different embedding media. Especially for the implementation of electronic devices to determine the working length in a university teaching scenario^25–27^, the question of which embedding media is most suitable not only for teaching purposes but also for research arises and, according to the actual specific literature, this matter is not completely elucidated. Thus, the aim of this study was to compare five different embedding media with an ex vivo research model and to establish if all the investigated embedding media provide similar accuracy results when determining the working length—a result which would consequently enhance educational and investigative confidence. The null hypothesis stated that the electronic working lengths measured with the embedding media investigated would result within a tolerance range of ± 0.5 mm. To reject this hypothesis, an ex vivo study was designed and carried out to assess if one or more of the embedding materials investigated would not be suitable conductive media for apex locator working length determination.

## Materials and methods

A total of 110 single-rooted human permanent teeth with mature apices were collected from an oral surgery department of a German university dental school for reasons (usually for periodontal, endodontic, orthodontic and traumatic reasons) unrelated to this investigation and included in this study. This research material can be considered as so-called excess material, and hence fulfills the legal regulations of the University Medical Center of the Johannes Gutenberg University of Mainz, Germany (Contract General Terms [AVB], §14 Organ explantation/further use of body material, Status: 1. April 2017) and may be used for medical research without any additional approval of the local ethics committee. This regulation is supported and approved by the ethics committee of the Medical State Association of Rhineland-Palatinate, Germany, for scientific purposes. Informed tooth extraction and further investigative purposes with the excess material consent was obtained from each individual. All methods were performed in accordance with the guidelines and regulations and experimental protocols at the University Medical Center of the Johannes Gutenberg University of Mainz, Germany.

Selection criteria were: complete root development, no signs of root fracture or resorption, no radicular or coronal caries, no partially or completely obliterated root canals and no previous endodontic treatment. In order to dissolve any superficial remaining tissue, the teeth were stored in a 1% sodium hypochlorite (Apotheke der Universitätsmedizin Mainz, Mainz, Germany) solution for 14 days. The teeth surfaces were thoroughly cleansed from tissue and calculus residue with an ultrasonic device (Piezon 150; EMS, Nyon, Switzerland). The teeth crowns were then separated at the enamel-cement interface with a 2-N feed force and a grain-size D64 diamond-coated cutting belt (EXAKT 300 CL; Exakt Advanced Technologies, Norderstedt, Germany) transverse to the tooth longitudinal axis; thus, defining a leveled and reproducible reference landmark. The length between this landmark and the corresponding physiological foramen was defined as the actual working length. Root canal patency was confirmed with a K-file 06 (Flexofile; Dentsply, Ballaigues, Switzerland) and the teeth fixed in Nalgene tubes (Nalgene; Rochester, NY, USA) with plaster enabling a direct contact between the root(s) and the embedding media. After root canal patency verification, no further irrigating or preparation procedures were undertaken, thus the electronic measurements were made under a relative low humidity in the root canals. The root canal entries were blocked with wax plates (Pinnacle Modellierwachs Standard; Dentsply De Trey, Konstanz, Germany) to prevent plaster from flowing into the root canals. The tubes were numbered consecutively.

Five embedding media were investigated: alginate (Blueprint; Dentsply De Trey, Konstanz, Germany), stick sponge (Steckschaum; Blume 2000, Norderstedt, Germany), 2% agar–agar (Becton Dickinson, Sparks, MD, USA), bovine skin 6% gelatin and 12% gelatin (Bovine gelatin; SIGMA, Steinheim, Germany). A 20 ml syringe (ECOJECT; Dispomed Witt, Gelnhausen, Germany) was then filled with the alginate, using a cement spatula. The Nalgene tubes were then injected with moderate pressure and a vibrator, taking care to prevent air inclusions and allowing a homogenous alginate filling of the tubes. Directly afterwards, the tube lids with the fixed roots were screwed on tightly, allowing maximum surface contact between the roots and the embedding media. The alginate was allowed to set for 2.5 min at 23 °C. A 0.5 cm length and 0.5 cm diameter stick sponge was cut off from a sponge stick, inserted into the Nalgene tubes and trimmed according to the respective tube length. The sponge was then moistened using a 10 ml syringe (ECOJECT; Dispomed Witt, Gelnhausen, Germany) and 0.9% saline solution (Bacto Agar; Becton Dickinson, Sparks, MD, USA) until the entire sponge was soaked. The agar–agar solution was prepared from 2 g agar–agar powder (Bacto Agar; Becton Dickinson, Sparks, MD, USA), 0.9 g sodium chloride, 0.095 g disodium hydrogen phosphate dehydrate, 0.018 g potassium dihydrogen phosphate (Optipur; Merck, Darmstadt, Germany) and 100 ml distilled water (Aqua B. Braun; B. Braun Melsungen, Melsungen, Germany). The mixture was heated to 150 °C under constant stirring and boiled until a homogeneous agar–agar suspension was formed. The Nalgene tubes were completely filled with the agar–agar solution by means of a disposable 10 ml pipette (Eppendorf Research Plus 10–100 µl; Eppendorf, Hamburg, Germany), the lids with the fixed roots were screwed on tightly and the solution, of gel-like consistency, was allowed to cool. The 6% and 12% gelatin solutions were prepared with 6 and 12 g gelatin, respectively, and a 100 ml 0.9% saline solution. The solutions were heated at a slowly increasing temperature rate up to 150 °C for 15 min and stirred continuously; they were subsequently allowed to cool and solidify.

**Figure 1:**
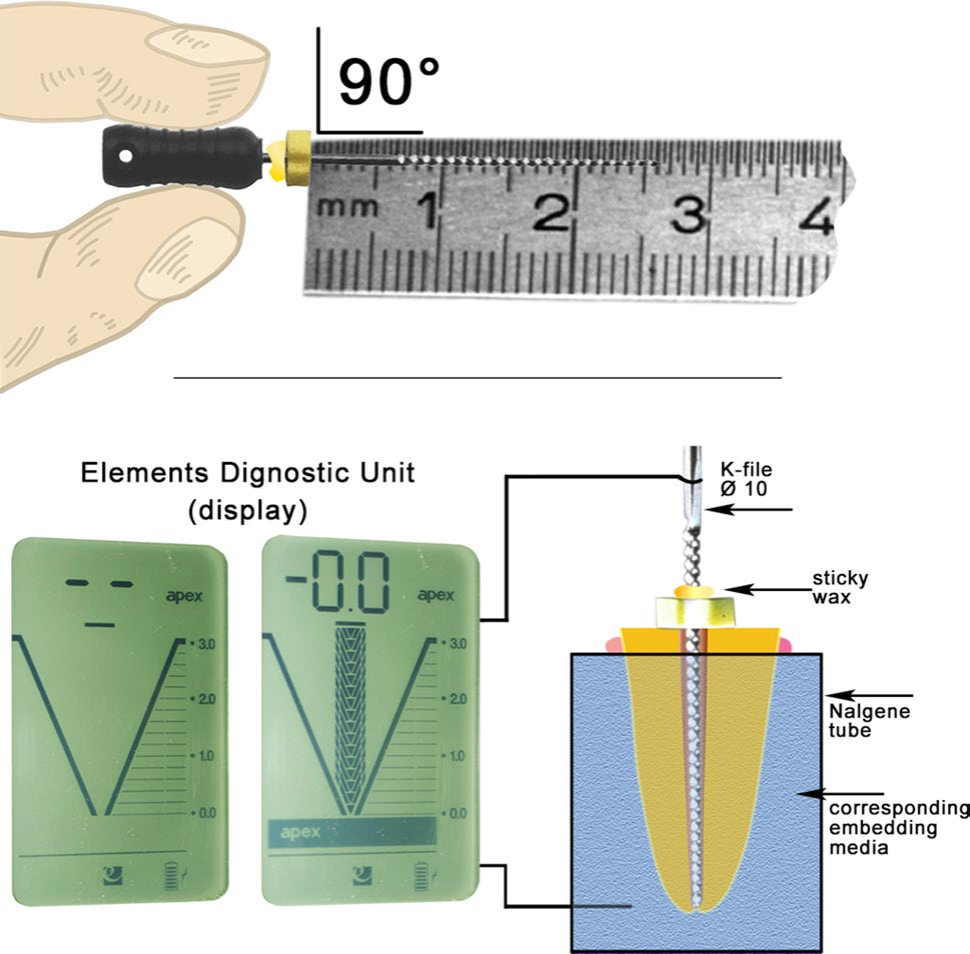
Diagrammatic visualization of the experimental setup of the electronic working length measurements (below) and precautions taken during the transmission of the corresponding measurements made (above). The Elements Diagnostic Unit apex locator measurement was determined to be correct as the display scale reached the “0.0” level, the “apex” sign below it appeared and a corresponding acoustic signal was heard.

All electronic measurements were carried out with the Elements Diagnostic Unit apex locator (Kerr, Brea, CA, USA) immediately after the corresponding preparation procedures were completed and within a time period of at most 15 min; otherwise, a new embedding media would have to be prepared. In accordance with the unit operating instructions, the measurements were carried out when the unit battery was sufficiently charged and after checking proper functioning of all cables and plug connections. A series of ex vivo measurements with teeth not included in this investigation were made under magnification (16 × ; Stemi DRC; Carl Zeiss Jena, Jena, Germany) until the working length with the Elements Diagnostic Unit apex locator at the physiological foramen was determined to be accurate. The electronic measurements were determined to be correct as the device display scale reached the “0.0” level, the “apex” sign below it appeared and a corresponding acoustic signal was heard (Fig. 1). The loop-shaped electrode, which is usually placed in the corner of the patient's mouth and establishes contact with the cheek, was replaced by a 1.7 mm Ø, 30 mm length stainless-steel wire (Stainless steel hard rods—316–1.70 mm/0.0393 inch; Sadevinox; Seynod, France) during the research procedures. The wire fitted tightly into the device connection socket and into a circular perforation made on the corresponding Nalgene tube. This perforation was made in the lower third of each Nalgene tube with a red round diamond bur (016; Premium Diamantschleifer; Busch & CO., Engelskirchen, Germany). The stainless-steel wire was glued (Supergel Sekundenkleber; UHU, Bühl, Germany) to the Nalgene tube in order to firmly affix the wire and to prevent the embedding media from flowing out through the perforation (Fig. 1).

All electronic measurements were carried out with K-files ISO 10 (Flexofile; Dentsply, Ballaigues, Switzerland), whereby the file was gently advanced towards the root tip until the apex locator showed a stable reading at the physiological foramen for five seconds. After having reached the working length, the silicone stopper was placed flat on the coronal root reference landmark, fixed on the K-file (Sticky Wax; Kerr, Brea, CA, USA) and the K-file was removed from the root canal carefully to ensure that the stopper position was not modified. Subsequently, the measured working length was determined with a 15 cm stainless-steel ruler (Rumold, Stuttgart, Germany) with half millimeter marks (± 0.2 mm) by an experienced and calibrated single operator (Fig. 1). Care was taken to ensure that the root surfaces were clean from any embedding media prior to any new working length measurement. After the working lengths were determined with the five different embedding media, the actual working lengths were determined as the tip of the measuring instrument reached the physiological foramina under direct view with a stereo microscope (16 ×) by one previously calibrated operator (A.M.) with an experience of over 100 physiological foramina localizations and according to a previously reported physiological foramina morphological description^3^.

The actual working length was established as a reference measurement for the purpose of comparison and to investigate the accuracy of the results obtained with the different embedding media. The working length of all root canals was determined with each embedding media (n = 550); thus, a total of 660 measurements (including the actual working length measurements) were made. The statistical evaluation was carried out with SPSS 15 Statistics Software (IBM; Armonk, NY, USA) and the Institute for Medical Biometry, Epidemiology and Informatics (IMBEI) at the University Medical Center facilities in Mainz, Germany. The results of the different embedding media were compared with the reference measurements of the actual working length obtained under the microscope. A measurement difference between the electronic and actual working lengths of ± 0.5 mm was defined as clinically acceptable. The absolute and relative frequencies of the results, measurements within the acceptable and non-acceptable tolerance ranges and with a significant difference from the reference measurements were calculated for each embedding media and graphically displayed using histograms and box plots. The Wilcoxon test for paired samples (α = 0.050) was bilaterally calculated and therefore considered as exploratory.

## Results

The working length results of 110 root canals measured with the Elements Diagnostic Unit apex locator and five different embedding media (alginate, sponge, 2% agar–agar, 6% and 12% gelatin) can be considered as normally distributed; thus, the results are described with mean values and confidence intervals. The absolute and relative results of the actual working length and different embedding media are given in Table 1. Only one significant difference between the actual and alginate root canal working lengths, when not taking into consideration the ± 0.5 mm clinical tolerance, could be observed (p = 0.035). The results obtained with all embedding media delivered homogeneous results. The descriptive statistic of the results showed that the mean values of all measurements ranged between 12.98 mm (alginate) and 13.15 mm (sponge) with the exception of 2% agar–agar and 6% gelatin, where a minimum of 9.5 mm and 10.5 mm, respectively, was measured, whereas all other embedding media showed a minimum of 10.0 mm. A maximum of 17.0 mm was obtained with all embedding media. The standard deviation ranged from 1.48 for 6% gelatin and 1.68 for sponge (Table 2).

**Table 1:** Actual working length and different embedding media working lengths determination (mm) distributed according to the corresponding root canal working lengths measured (WL).

WL	Actual		Alginate		Sponge		2% agar–agar		6% gelatin		12% gelatin	
	n	%	n	%	n	%	n	%	n	%	n	%
9.5							1	0.9				
10.0	1	0.9	2	1.8	1	0.9	3	2.7			2	1.8
10.5	6	5.5	12	10.9	7	6.4	3	2.7	6	5.5	6	5.5
11.0	6	5.5	6	5.5	10	9.1	9	8.1	9	8.2	9	8.2
11.5	13	11.8	7	6.4	10	9.1	14	12.7	10	9.1	8	7.3
12.0	9	8.2	9	8.2	5	4.5	5	4.5	7	6.4	10	9.1
12.5	10	9.1	9	8.2	13	11.8	9	8.2	10	9.1	8	7.3
13.0	14	12.7	13	11.8	12	10.9	15	13.6	17	15.5	14	12.7
13.5	16	14.5	15	13.6	12	10.9	14	12.7	15	13.6	15	13.6
14.0	11	10.0	14	12.7	12	10.9	11	10.0	13	11.8	14	12.7
14.5	10	9.1	9	8.2	9	8.2	11	10.0	10	9.1	10	9.1
15.0	6	5.5	6	5.5	6	5.5	6	5.5	5	4.5	5	4.5
15.5	4	3.6	5	4.5	6	5.5	4	3.6	4	3.6	6	5.5
16.0	1	0.9	1	0.9	1	0.9	2	1.8	1	0.9		
16.5	2	1.8	1	0.9	4	3.6	2	1.8	2	1.8	1	0.9
17.0	1	0.9	1	0.9	2	1.8	1	0.9	1	0.9	2	1.8
Total	110	100.0	110	100.0	110	100.0	110	100.0	110	100.0	110	100.0

Significant higher differences between the results obtained with alginate and the actual working lengths are noticeable. However, it should be taken into consideration that the clinically acceptable tolerance of ± 0.5 mm accepted in this investigation, is not being taken into account in this table (n = 110 per research group).

**Table 2:** Statistical evaluation of the working length determination with the Elements Diagnostic Unit apex locator and five different embedding media (mm; MED = median, 95% CI = 95% confidence interval of the difference; SD = standard deviation; n = 110 per research group.

	n	Min	Max	Mean	MED	95% CI	SD
Alginate	110	10.0	17.0	12.98	13.0	12.68–13.28	1.59
Sponge	110	10.0	17.0	13.15	13.0	12.83–13.47	1.68
2% agar–agar	110	9.5	17.0	13.05	13.0	12.74–13.35	1.60
6% gelatin	110	10.5	17.0	13.09	13.0	12.81–13.37	1.48
12% gelatin	110	10.0	17.0	13.07	13.0	12.78–13.37	1.55

**Figure 2:**
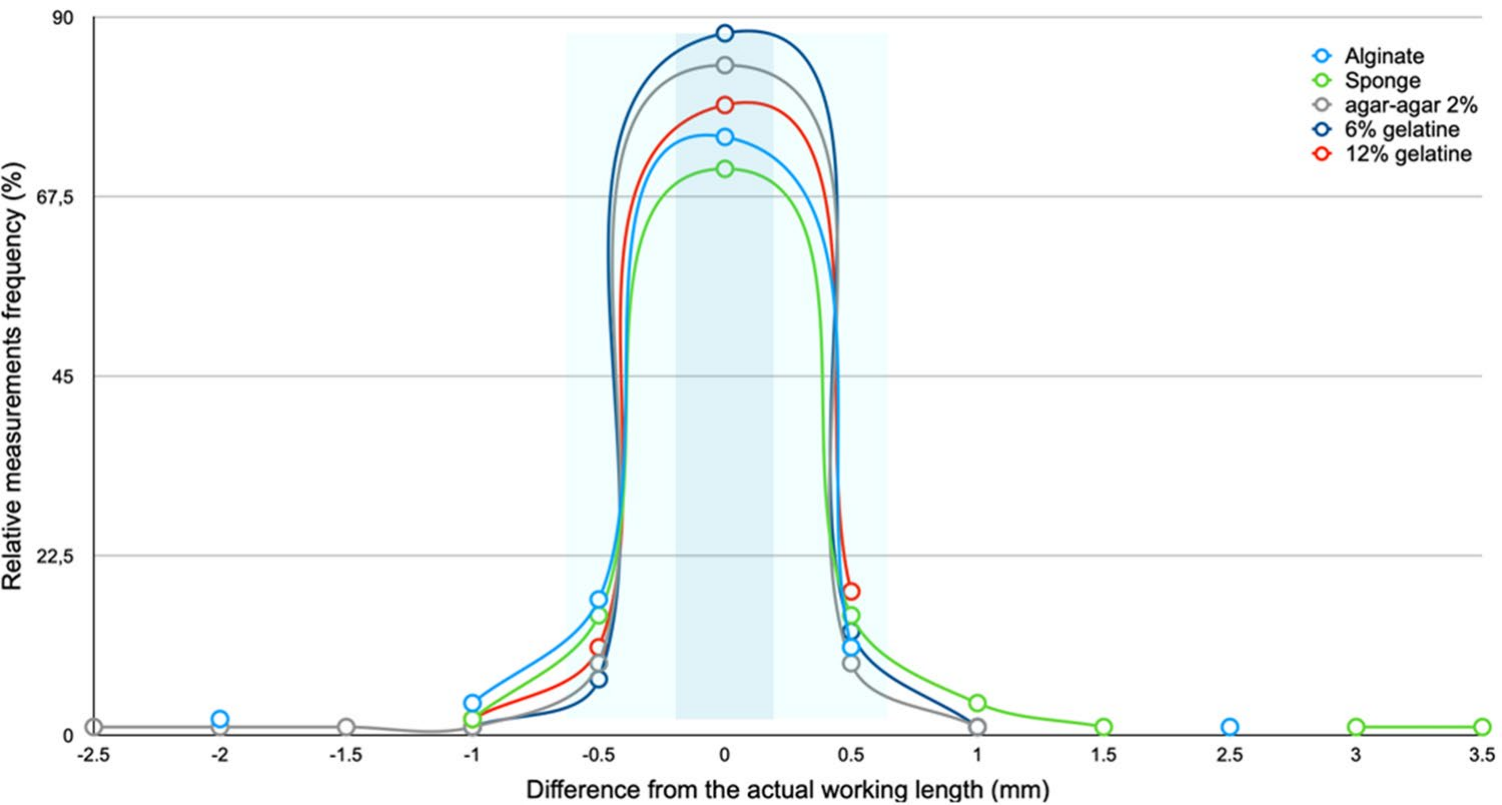
Diagram depicting the relative frequency (%) differences of the working length measurements obtained with the different embedding media and the actual working length of the root canals investigated. A ± 0.5 mm difference was considered in this investigation as clinically acceptable (the exact and clinically acceptable measurements are highlighted with dark and light blue, respectively; n = 110 per research group).

**Figure 3:**
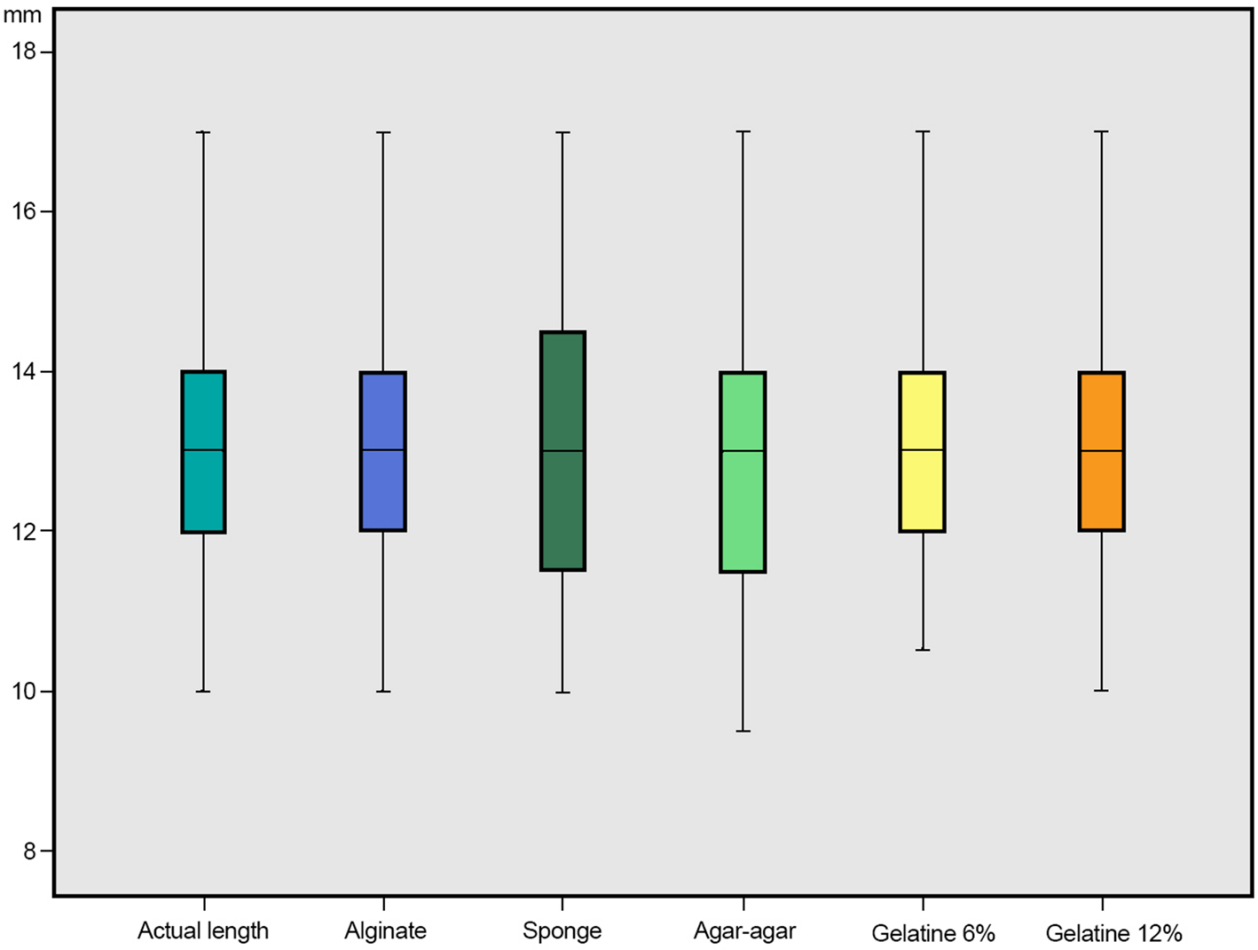
100% box-and-whisker plot of the working length determination measurement results with the different embedding media investigated (mm; n = 110 per research group).

**Figure 4:**
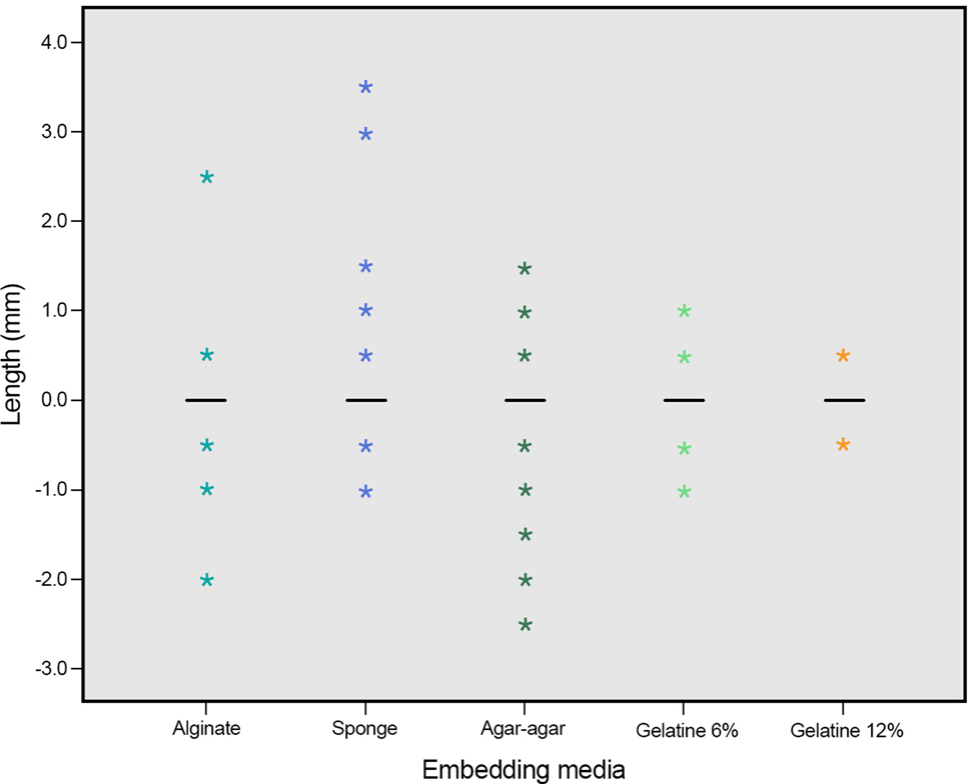
Box plot showing the differences distribution between the actual working length (0.0) and the ones obtained with the different embedding media. The lines at “0.0” represent the 25th quartile, median and 75th quartile. Consequently, a characteristic box cannot be seen in the present figure. A slight tendency of too long and too short measurements for sponge and agar–agar, respectively, can be observed. For alginate and gelatin in both concentrations the scattering is similar (mm; n = 110 per research group).

Only few shorter and longer measurements within the non-clinically acceptable working length were observed. Overall, 10 measurements 1.0 mm shorter than the working length (4 = alginate, 2 = sponge and 12% gelatin and 1 = 2% agar–agar and 6% gelatin) and five measurements 1.5 mm to 2.5 mm shorter than the working length were observed (2 = alginate and 3 2% agar–agar). Six measurements 1 mm longer than the working length (4 = sponge and 1 = 2% agar–agar and 6% gelatin) and five measurements 1.5 mm to 3.5 mm shorter than the working length were observed (1 = alginate and 4 sponge). The high accuracy of measurements obtained at the physiological foramen and within the ± 0.5 mm clinical tolerance can be observed in Fig. 2. The results range and individual boxes show overall a relative uniformity of the working length measurements made with the different embedding media. It could be observed that the largest measurement at 17.0 mm and the median position at 13.0 mm are very similar for all embedding media (Fig. 3). The differences between the electronically determined working lengths in the respective embedding media and the actual working lengths are shown in Fig. 4.

## Discussion

The working length results of 110 root canals at the physiological foramen level determined by using the Elements Diagnostic Unit apex locator and alginate, sponge, 2% agar–agar, 6% and 12% gelatin used as embedding media do not allow to reject the null hypothesis. Moreover, in accordance with several reports^22,28^ a clinically acceptable tolerance range for working length determination accuracy at ± 0.5 mm was also allowed in this investigation. Whereas a relatively high accuracy of working length determination with the radiographic method has been reported with an ex vivo research model^16^, it should be taken into account that, within this type of research set-up, the bone structures are not depicted, which could clinically represent a burdensome clinical situation when detecting meticulous endodontic areas, particularly for operators with little experience. Thus, it seems reasonable to permit a working length determination accuracy tolerance range due to the fact that an exact morphological^3^ or radiological^29–31^ determination of the physiological foramen is not possible in daily practice. When considering the clinical implication of these two parameters (working length determination method and clinical tolerance), a tolerance allowance can also be supported based on a radiological investigation^1^ in which the authors report a 95% success rate for endodontic treatments.

The morphological landmarks of the apical region illustrate a particular terminology problem in scientific research. Theoretically, the terminus of a root canal is where the pulp tissue or dentin root canal comes apically to an end. This landmark has been recommended as the terminus of the root canal shaping and obturation procedures^4,32^. Consequently, this landmark is where the working length should be determined by means of either a radiograph or an electronic device or a combination of both methods^14,15,33^. Typically, the root canal narrows consistently from coronal expanding apically to form the physiological foramen (apical constriction)^3,34^. The major (apical) foramen is considered to be located at the root surface, whereas the physiological foramen (minor foramen; apical constriction) is considered to be the narrowest (minor) diameter of the root canal located at the cementodentinal junction approximately 0.5 to 1 mm away from the radiological apex^34^. However, it has been reported that an apical constriction was observed in less than 50% of the teeth investigated^35^ and that the cement root canal area has not only tapered walls but also parallel walls^36^. Furthermore, it has also been reported that the physiological foramen cannot always be clearly ex vivo delimited under magnification (40 ×)^3^; yet, it can be delimited when being investigated under micro-CT^37^.

In morphological and working length investigations, the physiological foramen has been termed as “apical constriction”^17,19,21,23,27,30–32,38–41^ “anatomic apex”^42,43^, “1 mm from the anatomical foramen”^44^, “minor foramen”^38–40,45^, “flush with the apex”^46^, “histological foramen”^34^, among others. The term physiological foramen has been proposed to define this morphological landmark^3^ since this landmark is invariably located at the junction between the pulpal connective and interstitial loose connective tissues of the periodontal ligament independently if an apical constriction is assuredly present or if its shape is conical or parallel. The accuracy range of electronic apex locators has been reported to be contrasting^21,38–40^. This wide accuracy range might also be explained due to the apical morphological landmarks terminology consistency lack within the reported investigations. In this investigation the positions of the physiologic foramina were determined through ex vivo observations under magnification (16 ×) made by one operator and a corresponding “0.0” reading of the electronic device employed. Although in the pertaining Elements Diagnostic Unit apex locator user’s manual^47^ the physiological foramen is termed as “apical foramen”, the highly accurate measurements results obtained in this investigation strongly suggest that the term *apical foramen* corresponds with physiological foramen.

This ex vivo investigation examined the accuracy influence of different embedding media on endodontic working length determination and an electronic device (Elements Diagnostic Unit) routinely used in clinical practice. In order to reproduce a clinical situation as closely as possible, an embedding media with electrical conductivity similar to that of the human periodontal tissue is indispensable in ex vivo investigations. A 2% phosphate-buffered agar–agar solution to mimic the periodontium conditions was proposed^48^. The advantage of this medium is that an electrical resistance of 6.5 kΩ with a 7.3 pH corresponds with the values of human periodontal tissue. Other reports^10^ have used the same agar–agar solution in their experiments, or with a 1% concentration^49^. The use of a 0.9% isotonic saline solution^41^ and gelatin^50^ as embedding media for ex vivo electronic working length determination experiments has also been also reported. Two different gelatin concentrations were included in this research in an effort to prove if possible different fluidities could have an impact on the electronic working length measurements, specifically in an educational scenario where the time required to make a measurement could be inherently extensive. Measurements using sponges or gels soaked in isotonic saline solution have been suggested as a further alternative method^51^. The working length accuracy results obtained in this research range between 91.7% (sponge) and 98.2% (gelatin 6% and 12%) and are similar to the ones reported by other investigation groups with research on different embedding media^9,21,49^. The use of sponges soaked in isotonic saline solution and gelatin has also been investigated^52^ and, similar to the results of this investigation, no statistically significant differences were reported. Several studies^8,9,16,21,23,27,53^ have compared the accuracy of different apex locators in vitro and alginate as embedding media and report accuracies ranging from 31 to 100%. However, a direct comparison of these results is cumbersome, mainly as a consequence of the different individual parameters investigated. In a systematic review and literature meta-analysis, the authors^19^ conclude that the precision of electrical length measurement thus depends on both the device and the type of irrigation.

Different research groups^53–55^ have dealt with the influence of different embedding media on the measurement accuracy of electronic apex locators. Successive measurements in 1% agar–agar, alginate, gelatin and 0.9% saline and flower sponge as embedding media and the Root ZX apex locator were investigated. These authors^54^ report that flower sponge was the only embedding media in which the working length was determined beyond the apical limit in 20% of cases; however, no statistically significant differences between the embedding materials were observed. In the present study, the longest measurements, which were up to 3.5 mm beyond the actual working length and thus clearly far beyond the clinically acceptable tolerance, were also obtained with sponge sticks. A possible explanation for these long measurements could be that sponge is easily deformable and therefore an unstable contact between the root apical region and embedding media is a given.

Alginate could be considered as a widespread embedding media in ex vivo endodontic apex locator research^16,22,23,53–55^. In contrast with this investigation, in which the only statistically significant difference working length accuracy measurement was determined for alginate (6 = shorter and 1 = longer), in a different investigation^54^ it has been reported that the most accurate measurements obtained were with alginate; however, this was when the file tip was at most 1 mm away from the “apical foramen”. The significant differences obtained in this investigation could be explained by arguing that the after-setting time of alginate could influence the contact between sample and embedding media. Although it could be postulated that the lower accuracy of the alginate media has no clinical significance, the possibility that a defective contact between the embedding media and sample could happen should be taken into consideration within an educational scenario in which time plays a decisive role. A different research group^55^, using a Root ZX II device, report that alginate has a higher electronic root canal length determination accuracy when compared with saline, and floral foam and gauze both soaked in 0.9% saline. In another similar investigation, the authors^53^ using the Raypex 5 and Dentaport ZX, report that alginate at a 0.5 mm and 1.0 mm tolerance showed the highest accuracy when compared with sugar-free gelatin and a 0.9% sodium hypochlorite solution; however, the authors do not report any statistically significant differences. This is in contrast to this investigation in which the results showed exact measurements in 64.5% = sponge, 68.2% = alginate, 71.8% = 12% gelatin, 76.4% = 2% agar–agar and 80% = 6% gelatin. In addition when a clinically acceptable tolerance (± 0.5 mm) was assumed, the range of correct measurements obtained rose to 91.7% = sponge, 92.8% = 2% agar–agar, 93.7% = alginate and 98.2% = 6% and 12% gelatin. These differences could be explained through the employment of different root canal irrigation research parameters and through the different clinical accuracy tolerances allowed in both investigations.

25 years ago, electronic apex locators were seldom routinely employed^7^ in daily practice, most probably due a lack of trust in the electronic devices on the side of the operator and to high interference susceptibility in older generation devices. However, nowadays the use of electronic apex locators in daily practice has markedly increased^5,6^ since actual working length determination devices have proven to be highly reliable in moist root canals^9,19,22^, in root resorption^13^, root fracture^11^ and during endodontic re-treatment cases^12^. Yet, the possibility of an incorrect measurement with an electronic working length determination device, even if these occur relatively seldom, should always be kept in mind. Therefore, a combined electronic and radiographic working length determination is to be preferred. The implementation of cone-beam computed tomography (CBCT) to determine the working length has been also discussed^56^; however, at least for the time being, CBCT cannot be recommended as a primary working length determination method. Under the presumption that a combined, educated clinical deployment strategy of an electronic device and invasive methods during the working length determination will enhance the working length determination accuracy, the ALARA principle (“as low [radiation] as reasonably achievable”) should always be taken into consideration^16,18^. The accuracy of different electronic devices has been extensively discussed. In fact, nowadays the use of an electronic apex locator and a radiograph is actually recommended by different professional endodontic societies^14,15,33^ as a routine clinical procedure to determine working length. The authors completely agree with this clinical guideline. Further discussion of this clinical aspect is beyond the scope of this research, namely the accuracy of different embedding media in endodontic working length determination for investigative and educational purposes. Although the results obtained in this research cannot be considered to refute the null hypothesis and the accuracy of alginate as an embedding media proved to be high, it should be kept in mind that this type of embedding media could be negatively influenced by the material manipulation time, especially within an educational scenario.

## Conclusions

According to the results obtained and within the limitations of this study, it can be concluded that all investigated embedding media are suitable for ex vivo endodontic investigative and educational purposes.
